# The Role of Body-Related and Environmental Sources of Knowledge in the Construction of Different Conceptual Categories

**DOI:** 10.3389/fpsyg.2012.00430

**Published:** 2012-10-29

**Authors:** Guido Gainotti

**Affiliations:** ^1^Center for Neuropsychological Research, Università Cattolica del Sacro Cuore, Policlinico Universitario Agostino GemelliRome, Italy; ^2^Department of Clinical and Behavioral Neurology, Istituto di Ricovero e Cura a Carattere ScientificoFondazione Santa Lucia, Rome, Italy

**Keywords:** models of conceptual knowledge, category-specific semantic disorders, animals vs. fruits and vegetables, sources of knowledge, anterior temporal lobes, left fronto-parietal lesions

## Abstract

Controversies exist regarding: (a) the relationships between perceptual and conceptual activities and (b) the format and neuro-anatomical substrates of concepts. Some authors maintain that concepts are represented in the brain in a propositional, abstract way, which is totally unrelated to the sensory-motor functions of the brain. Other authors argue that concepts are represented in the same format in which they are constructed by the sensory-motor system and can be considered as activity patterns distributed across different perceptual and motor domains. The present paper examines two groups of investigations that support the second view. Particular attention is given to the role of body movements and somatosensory inputs in the representation of artifacts and, respectively, of visual and other perceptual sources of knowledge in the construction of biological categories. The first group of studies aimed to assess the weight of various kinds of information in the representation of different conceptual categories by asking normal subjects to subjectively evaluate the role of various perceptual, motor, and encyclopedic sources of knowledge in the construction of different semantic categories. The second group of studies investigated the neuro-anatomical correlates of various types of categorical disorders. These last investigations showed that the cortical areas damaged in patients with a disorder selectively affecting a given category have a critical role in processing the information that has contributed most to constructing the affected category. Both lines of research suggest that body movements and somatosensory information have a major role in the representation of actions and artifacts mainly known through manipulations and other actions, whereas visual and other perceptual information has a dominant role in the representation of animals and other living things.

## Introduction

Our knowledge of the world is mediated by two types of activity: (1) perceptual-motor activity, which allows us to obtain information about external objects through our actions or analysis of the perceptual attributes of environmental stimuli; and (2) conceptual activity, which permits the construction of internal representations of complex categories of knowledge. Two points must be stressed regarding this basic distinction. First, objects we know mainly through actions accomplished by our body only partly overlap with those we principally know through auditory and visual modalities. The former usually belong to the category of artifacts that can be touched, manipulated and used for different purposes, whereas the latter often belong to living categories (such as wild animals) we know in a physical or virtual environment located in far extra-personal space. The second point refers to the fact that until recently there was an important gap between our knowledge of the mechanisms and neuro-anatomical substrates of perceptual and motor activities and respectively of conceptual activities. Indeed, we have clear and detailed knowledge about the organization and neuro-anatomical structures subsuming actions or different perceptual modalities, but we have only controversial and uncertain models about the format and neuro-anatomical substrates of concepts.

Concerning the format of conceptual representations, many cognitive models (e.g., Phylyshyn, 1973; Fodor, 1975 and, more recently, Humphreys and Riddoch, 1988; Riddoch et al., 1988; Caramazza et al., 1990; Patterson and Hodges, 2000; Coccia et al., 2004) have claimed that perceptual and conceptual activities result from the activity of interrelated but completely independent systems.

According to this view, the hierarchical stages of perceptual analysis proceed up to the level of structural description, which includes a complete three-dimensional specification of the sensory characteristics of objects. But after this level, no trace of the previous sensory-motor mechanisms persists because the format of conceptual representations accessed through these structural descriptions is considered abstract, amodal, and propositional. Nevertheless, subsequent to the pioneering work of Kolers and Brison (1984), Allport (1985), and Jackendoff (1987), an increasing number of authors have refuted this model of an abstract, amodal conceptual/semantic system. These authors maintain that conceptual representations keep the stamp of the perceptual mechanisms through which they were formed and are stored in the same format in which they were constructed. Drawing in part on these cognitive models and in part on Hebb’s (1949) model of “cell assemblies,” Damasio (1989, 1990) proposed the dynamic “higher-order convergence zone” construct. This construct assumes that concept retrieval results from a process of recollection of modality-specific bits of memories, stored near the sensory portals and motor output sites of the system and triggered by firing in “higher-order convergence zones” (Damasio’s, 1989, 1990). This construct was further developed by Barsalou (Barsalou, 1999; Barsalou et al., 2003), who added the similarity-in-topography (SIT) principle to Damasio’s model. According to this model, the proximity of two conjunctive neurons in a convergence zone increases with the similarity of the features they conjoin. Consequently, conjunctive neurons become topographically organized into local regions that represent properties and categories. Both Damasio’s and Barsalou’s models can be considered eminent examples of “embodied cognitive models,” whose central tenet is that semantic knowledge is grounded in sensory-motor systems that are automatically engaged during online conceptual processing, re-enacting modality-specific patterns of activity normally evoked during perception and action.

### Summary of the main points of this section

The format of conceptual representations is the object of a strong debate between authors who maintain that concepts are represented in a propositional, abstract manner in the brain and authors who argue that concepts are represented in the same format in which they are constructed by the sensory-motor system. Some models are discussed that specify how sensory-motor information could converge in the construction of a conceptual representation.

## The Discovery of Category-Specific Semantic Disorders and Their Impact on the Debate between Supporters of the Abstract and the Embodied Format of Conceptual Representations

One important development in the debate between supporters of the “abstract/amodal” and “embodied/sensory-motor” format of conceptual representations was the discovery that disruptions of conceptual knowledge are not necessarily homogeneous across categories but are sometimes “category-specific” (Warrington and McCarthy, 1983, 1987; Warrington and Shallice, 1984). Category-specific semantic disorders usually affect biological (“living”) more than artifact (“non-living”) categories, but sometimes preferentially impair artifact (“non-living”) categories (see Saffran and Schwartz, 1994; Gainotti et al., 1995; Gainotti, 2000, 2005; Capitani et al., 2003 for reviews). In any case, they have been explained differently by supporters of the abstract and the embodied format of conceptual representations.

In particular, Warrington and co-workers (Warrington, 1975, 1981; Warrington and McCarthy, 1983, 1987; Warrington and Shallice, 1984) claimed that their patients’ semantic disorders did not respect the boundaries between living/biological and non-living/artifact entities (e.g., the representation of “body parts” tended to be disrupted together with that of artifact categories, whereas the representation of “musical instruments” tended to be disrupted with that of living items). This suggested that “category-specific semantic disorders” might not be due to the disruption of true “biological” and “artifact” categories, but might be the by-products of a more basic dichotomy concerning the differential weighting of visual-perceptual and functional attributes in the representation of biological and, respectively, artifact categories. This interpretation is obviously at variance with the views of authors who claim that the format of conceptual representations is abstract, amodal, and propositional. In fact, these authors (e.g., Caramazza, 1998; Caramazza and Shelton, 1998; Capitani et al., 2003; Caramazza and Mahon, 2003) proposed a very different theoretical interpretation of category-specific semantic disorders. They hypothesized an “innate” categorical organization of conceptual knowledge in which category-specific impairments for animals, plant life and artifacts are due to the disruption of innate brain networks shaped by natural selection for rapid identification of objects that are very important for survival. This interpretation is more consistent with the model of an abstract, propositional semantic system (because all the above-mentioned categories could be represented in the same abstract format) but fails to explain the joint breakdown of artifacts with body parts (see McCarthy, 1995; Gainotti, 2000, 2004; Hart and Kraut, 2007 for reviews) and musical instruments with living items (see Dixon et al., 2000; Gainotti, 2000; Masullo et al., 2012 for reviews).

The present review examines two different groups of investigation that support the “embodied/sensory-motor” model of conceptual representations, devoting particular attention, on one hand, to the role of body movements and somatosensory inputs and, on the other hand, to that of visual and other perceptual sources of knowledge in the construction of different semantic categories.

The first group includes studies that evaluated the weight of various kinds of information in the representation of different conceptual categories by asking normal subjects to subjectively evaluate the role of various perceptual, motor, and encyclopedic sources of knowledge in constructing different living and artifact categories.

The second group includes studies concerned with the neuro-anatomical correlates of various types of category-specific disturbances, because it is important to check for consistency between the cortical areas damaged in patients with a disorder selectively affecting a given category and the specific functions these areas have in processing information that contributes to the construction of the affected category. In order to facilitate the comprehension of these rather hard issues, Table 1, reporting an overview and clustering of the main findings of the present survey has been included.

**Table 1:** **Overview of the main findings of the present review**.

Aims	To survey two different groups of investigations supporting the view that concepts are represented in the brain in the same format in which they are constructed by the sensory-motor system.
	The first group includes studies in which normal subjects were asked to subjectively evaluate the relevance of various perceptual, motor, and encyclopaedic sources of knowledge in the construction of different living and artefact categories.
	The second group includes studies that investigated the neuro-anatomical correlates of category-specific disorders for various types of living and artefact categories. The aim of these investigations consisted in assessing if the cortical areas damaged in patients with a disorder selectively affecting a given category have a critical role in processing the information that mainly contributed to the construction of that category.
Results of the first group of investigations	Studies consistently show: (a) that visual information is evaluated as the dominant feature in both living and artefact categories; and (b) that the next most relevant source of information consists of other perceptual data for the biological categories and of body-related features (i.e. actions and somatosensory data) for the artefact categories.
	These data suggest that the greatest difference between living and non-living categories is not the prominent role played by vision in the representation of biological entities and functional features in the representation of artifacts. Instead, the greatest difference is in the interaction between visual data and other perceptual attributes in the case of living beings and between visual data and action-related properties in the case of artifacts.
Results of the second group of investigations	These studies show that in patients with a category-specific semantic disorder for biological entities, lesions bilaterally affect the anterior parts of the temporal lobes (where the ventral stream of visual processing converges with auditory, olfactory, and gustatory inputs).
	On the contrary, in patients with a preferential impairment of the artifact categories lesions usually affect the left-sided fronto-parietal, sensory-motor cortices (where the dorsal stream of visual processing converges with body-related and action-oriented structures).
	Taken together, both lines of research suggest that body movements and somatosensory information have a major role in the representation of artifacts, whereas visual and other perceptual information have a dominant role in the representation of animals and other living entities.
Conclusion	The principle assuming that concepts are represented in the brain in the same format in which they are constructed by the sensory-motor system is consistent with the subjective evaluation of normal subjects and the main functions of the cortical areas affected in patients with disorders specifically affecting these conceptual categories.

### Summary of the main points of this section

The discovery of category-specific semantic disorders for living things and artifacts has strongly influenced the debate between supporters of the abstract and the sensory-motor format of conceptual representations. Warrington and co-workers maintained that category-specific semantic disorders were not due to the disruption of true “biological” and “artifact” categories but were the by-product of a more basic dichotomy concerning the different weight of visual-perceptual and functional attributes in the representation of biological and, respectively, artifact categories. Supporters of the abstract models counter-argued that category-specific impairments for animals, plant life and artifacts are not due to the loss of specific clusters of sensory-motor information but reflect an innate categorical organization shaped by natural selection to support rapid identification of objects important for survival. The present review examines two groups of investigations that support the sensory-motor model of conceptual representations. It takes into account, on one hand, studies conducted in normal subjects, to evaluate the weight of various kinds of information in the representation of different conceptual categories and, on the other hand, studies that investigated the neuro-anatomical correlates of various types of category-specific disorders.

## Studies that Assessed the Weight of Various Kinds of Information in the Representation of Different Conceptual Categories

The view that semantic knowledge is not stored in an amodal, abstract format in the brain, but in the same concrete format in which it was constructed by the sensory-motor system, was prompted by a series of seminal papers by Warrington and co-workers (Warrington, 1975, 1981; Warrington and McCarthy, 1983, 1987; Warrington and Shallice, 1984). These papers suggested (a) that different brain lesions can disrupt different categories of knowledge (e.g., living beings vs. artifacts); and (b) that these “category-specific disorders” are not due to the disruption of an innate categorical brain organization but to disorganization of the sensory-motor mechanisms that primarily contributed to the development of different categories (i.e., the “differential weighting hypothesis”). In particular, Warrington and Shallice (1984) described four patients recovering from herpes simplex encephalitis (HSE) who presented a dissociation between a selective impairment for living things and a relative sparing of artifacts. They believed that the dissociation was due to the major role of visual features in the identification of living things and functional features in the identification of artifacts. The lesions in HSE selectively affect the anterior parts of the temporal lobes, where the ventral stream of visual processing terminates (Ungerleider and Mishkin, 1982; Mishkin et al., 1984; Goodale et al., 1991). Therefore, Warrington and Shallice (1984) proposed that these structures have a critical role in the construction of living categories because they subsume the high-level visual data on which distinctions among members of the “living” categories are based. According to this view, the distinction between a lion, a tiger, and a leopard would depend on a visual sensory feature, namely, the plain, striped, or spotted aspect of their skin; in the case of artifacts, however, identification of a category member would depend on functional attributes (i.e., the subtly different functions man designed them for). This interpretation, which is usually called the “sensory-functional theory” (SFT; Caramazza and Shelton, 1998; Tyler et al., 2000; Capitani et al., 2003; Ventura et al., 2005), prompted studies in various areas of research in brain-damaged patients and normal subjects that reported conflicting results (see Capitani et al., 2003; Gainotti et al., 2009 for reviews). For example, the disproportionate impairment of visual (rather than functional) attributes predicted by the SFT in patients with a category-specific semantic impairment for living things was confirmed in some patients (e.g., Sartori and Job, 1988; De Renzi and Lucchelli, 1994; Gainotti and Silveri, 1996; Rosazza et al., 2003) but not in others (see Capitani et al., 2003 for survey). Moreover, the assumption of differential weighting of sensory and functional information in the representation of knowledge about living things and artifacts has not been systematically confirmed by studies conducted in normal subjects, using various experimental procedures, which will be described later.

These conflicting results are probably due to the inappropriateness of the expression “SFT” to account for the “differential weighting” hypothesis, because both “functional” and “sensory” features include very heterogeneous components. Based on the suggestion of Warrington and McCarthy (1987), Buxbaum et al. (2000), Buxbaum and Saffran (2002), and Boronat et al. (2005) distinguished, within the functional knowledge, the function of an object from its manipulation. They suggested that, because the “manipulation” is related to a sensory-motor activity, manipulation might be the component most tightly linked to the “differential weighting hypothesis”. The above-cited studies showed that also the properties subsumed by the term “sensory” are heterogeneous, because different types of sensory data might have different weights in the construction of different semantic categories. Thus, visual perception could have a leading role in the mental representation of animals and somatosensory data in that of tools. These facts prompted various authors (e.g., Gainotti, 1990, 2006; Saffran and Schwartz, 1994; Gainotti et al., 1995; Chao et al., 1999; Chao and Martin, 2000; Martin et al., 2000; Martin and Chao, 2001; Martin, 2007) to replace the expression “SFT” with the more specific “sensory-motor model of conceptual knowledge” (SMCK), which, in keeping with Barsalou et al.’s (2003) “embodied cognition theory,” assumes that various perceptual, motor and encyclopedic sources of knowledge have different weights in the construction of different living and artifact categories.

The assumption that various kinds of sensory information may have different weights in the representation of different categories of knowledge has been confirmed by studies conducted in normal subjects (following the principles of the SFT and the SMCK) to evaluate their mental representations of the sources of knowledge in these categories. Farah and McClelland (1991) and Caramazza and Shelton (1998) were the first authors who tried to assess the weight of various kinds of information in the representation of different conceptual categories in normal subjects. Following the principles of the SFT, they asked participants to underline either visual or functional descriptors in dictionary definitions of living things or artifacts. The results of these studies were conflicting. Farah and McClelland (1991) found a much larger ratio of visual than functional attributes for living things than for artifacts, whereas Caramazza and Shelton (1998) only found a non-significant difference between these two domains of knowledge. This discrepancy emerged because in the former study a property was considered “functional” only if it described “what the item did or what it was for,” whereas in the latter all “non-sensorial” (i.e., functional, encyclopedic, etc.) descriptors were contrasted with sensory properties. Analogous inconsistencies emerged in studies conducted by Devlin et al. (1998), Tyler et al. (2000), Garrard et al. (2001), McRae and Cree (2002), Vanovenberghe and Storms (2003), Ventura et al. (2005), and Zannino et al. (2006), when feature generation or feature verification tasks were used to check the assumption of differential weighting of sensory and functional information in the representation of knowledge about living things and artifacts.

Tranel et al. (1997b), Vigliocco et al. (2004), McRae et al. (2005), Gainotti et al. (2009), Hoffman and Lambon Ralph (under review), and Gainotti et al. (2012) obtained more consistent results using different procedures to test the principles of the “SMCK.” Tranel et al. (1997b) asked normal subjects who had been shown slides of entities from different conceptual categories to rate the extent to which a number of factors, including manipulability and various sensory modalities, had been part of their experience with the corresponding objects. Vigliocco et al. (2004) and McRae et al. (2005) gathered data on conceptual feature representations from the conceptual domains of objects and actions, by asking undergraduate students to list the features of the things the stimulus words referred to. They distinguished (in the object field) several categories of living things and artifacts and classified the features in five categories: visual, other perceptual, functional, action-related, and other (including superordinate and encyclopedic). Gainotti et al. (2009, 2012) and Hoffman and Lambon Ralph (under review) used a procedure that was more directly and specifically derived from the “SMCK.” This procedure consisted of asking normal subjects to use Likert scales to evaluate the influence of different perceptual (visual, auditory, tactual, olfactory, and gustative) and motor activities, as well as encyclopedic information, in the mental representation of living and artifact categories.

The same results were consistently found in all of these investigations using both feature-listing tasks and Likert scales to evaluate the weight of different “sources of knowledge.”

First, visual information was consistently evaluated with both methodologies as the dominant type of sensory or motor feature by averaging results obtained across all concepts and comparing the scores for each modality (Tranel et al., 1997b; Cree and McRae, 2003; Vigliocco et al., 2004; McRae et al., 2005; Gainotti et al., 2009; Hoffman and Lambon Ralph, under review). The major importance attributed to vision in the mental representation of all kinds of concrete entities is not surprising if we consider that most of our knowledge of the world is obtained through this perceptual modality.

Second, when hierarchical cluster analyzes were used in feature-listing studies (e.g., Cree and McRae, 2003; Vigliocco et al., 2004) or in studies based on a separate rating of the various sources of knowledge (e.g., Gainotti et al., 2009, 2012; Hoffman and Lambon Ralph, under review), a tripartite organization of knowledge (with three major clusters corresponding to animals, fruits and vegetables, and artifacts) was found.

Third, the distinction between living things and artifacts, on one hand, and “animals” and “plant life” (within the “living” categories), on the other hand, was confirmed by a more detailed analysis of the next most relevant sources of information after vision. In fact, the next most relevant sources of information consisted of other perceptual data (and encyclopedic information) for the living categories but of body-related features (actions and somatosensory data) for the artifact categories. Furthermore, within the “living” categories the next most relevant sources of information included encyclopedic knowledge and auditory perceptions (i.e., typical sounds) in animals, whereas they consisted of olfactory and gustatory perceptions and actions (e.g., peeling, cutting, and stirring) in fruits and vegetables.

Taken together, these data suggest that the greatest difference between living and artifact categories lies in the interaction between visual data and other perceptual (auditory, olfactory, gustatory, and tactual) attributes in the case of living things, and between visual data, action-related properties, and somatosensory information in the case of artifacts. The greatest difference between living and artifact categories does, therefore, not lie in the prominent role played by vision in the representation of animals, fruits, and vegetables, and by functional features in the representation of artifacts.

### Summary of the main points of this section

Warrington and co-workers’ “SFT” has not been systematically confirmed by studies conducted in normal subjects using various experimental procedures. Conflicting results may be due to the inappropriateness of the expression “SFT,” because both “functional” and “sensory” features include very heterogeneous components. Indeed, the expression has been replaced with the more specific “SMCK,” which assumes that various perceptual, motor and encyclopedic sources of knowledge have different weights in the construction of different living and artifact categories. The usefulness of this new model has been confirmed in studies performed to assess the weight of various kinds of information in the representation of different conceptual categories by asking normal subjects to subjectively evaluate the role of various sources of knowledge in the construction of different semantic categories. These studies have consistently shown: (a) that visual information is evaluated as the dominant feature in both living and non-living categories; (b) that the next most relevant sources of information are other perceptual data for the biological categories and body-related features (actions and somatosensory data) for the artifact categories.

## Investigations Concerning the Neuro-Anatomical Correlates of Various Types of Categorical Disorders

From the neuro-anatomical point of view, data obtained by studying evaluations of the weight of various kinds of information in the representation of different conceptual categories suggest that brain structures with a critical role in the representation of living and artifact categories might have a well-defined cortical localization. Thus, the anterior parts of the temporal lobes (where the ventral stream of visual processing converges with auditory, olfactory, and gustatory inputs) should have a critical role in the representation of biological entities. On the other hand, the fronto-parietal, sensorimotor cortices (where the dorsal stream of visual processing converges with body-related and action-oriented structures) should have a major role in the representation of artifacts. Furthermore, subjective evaluations of the weight of various kinds of information in the representation of different conceptual categories suggest there is a different degree of lateralization in the brain’s representation of animals, fruits and vegetables, and artifacts. The major sources of knowledge about animals (i.e., visual and auditory inputs) should, indeed, be bilaterally represented, whereas the action-oriented structures, which provide an important source of knowledge about artifacts (and to a lesser extent about fruits and vegetables), should be mainly represented in the left hemisphere, which controls the movements of the right side of the body.

Both of these predictions have been confirmed by a number of anatomo-clinical and neuroimaging studies.

Concerning the critical role played by lesions of the anterior parts of the temporal lobes in semantic disorders for biological entities, several reviews of the anatomical correlates of category-specific semantic disorders (e.g., Saffran and Schwartz, 1994; Gainotti et al., 1995; Damasio et al., 1996; Tranel et al., 1997a; Gainotti, 2000, 2005; Capitani et al., 2003) have shown that brain structures located in the terminal parts of the ventral stream of visual processing (such as the IT cortices) or responsible for integrating highly processed visual data with other sensory modalities (such as the perirhinal and entorhinal cortices) are usually disrupted in patients with category-specific semantic disorders for living things. For example, Gainotti (2000) made a detailed and systematic review of all available anatomo-clinical reports of patients who presented a category-specific semantic disorder for living things and artifacts and found bilateral injury to the antero-mesial and inferior parts of the temporal lobes (temporal pole, IT cortex, parahippocampal, perirhinal, and entorhinal cortices) in almost all patients with a category-specific semantic impairment for living things. Strauss et al. (2000) and Luckhurst and Lloyd-Jones (2001) also reported similar data, because they showed that temporal lobectomy patients were disproportionately more impaired in naming living than non-living things.

Data supporting this model were also reported by Grabowski et al. (2001), Devlin et al. (2002), Tyler et al. (2004), Moss et al. (2005), and Bright et al. (2005) in a series of neuroimaging studies. These authors showed that the human perirhinal cortex and neighboring anterior temporal structures provide the neural infrastructure for living categories.

For example, Devlin et al. (2002) entered data from seven PET studies into a single multifactorial design that crossed category (living vs. man-made) with a range of tasks and found that living things activated medial aspects of the anterior temporal poles bilaterally and tools activated a left posterior middle temporal region. And Bright et al. (2005) reviewed recent neuropsychological and neuroimaging studies which showed that the human perirhinal cortex and contiguous anteromedial temporal structures provide the neural infrastructure for making fine-grained discriminations among objects, suggesting that damage in the perirhinal cortex may underlie the emergence of category-specific semantic deficits for living things.

Regarding artifacts, we see that lesions of a network involving the dorso-lateral part of the left frontal lobe, the left inferior parietal lobe and the left middle temporal gyrus, where different components of action schemata are represented (see Saygin et al., 2004), provoke a prevalent impairment for tools and other man-made artifacts, whose knowledge is mainly based on active manipulation and physical contact with objects. This claim is supported by the results of Gainotti’s (2000) above-mentioned systematic review, which showed that an extensive lesion in areas located in the dorso-lateral convexity of the left hemisphere was present in all patients with a semantic impairment selectively affecting artifact categories, and by other more recent reviews (e.g., Capitani et al., 2003; Kellenbach et al., 2003; Gainotti, 2005; Buxbaum and Kalénine, 2010; Campanella et al., 2010).

The systematic restriction of brain lesions to the left hemisphere in patients with a category-specific disorder for artifacts was confirmed in activation studies, conducted by Chao and Martin (2000), Gerlach et al. (2002), Kellenbach et al. (2003), and Boronat et al. (2005), and in experiments of direct electrical cortical stimulation, conducted by Ilmberger et al. (2002). For example, Chao and Martin (2000) found that viewing and naming pictures of tools selectively activated the left ventral premotor cortex. Boronat et al. (2005) obtained similar results when participants viewed pairs of pictures or words denoting manipulable objects and had to determine whether the objects were manipulated the same way (M condition) or served the same function (F condition). Significantly greater and more extensive activations in the left inferior parietal lobe occurred in the M than the F condition. Finally, Ilmberger et al. (2002) used tool and animal items to test the naming capabilities of epilepsy patients with subdural electrodes implanted for localization of the epileptogenic zone and preoperative mapping of cognitive functions. Results showed that during stimulation of the left hemisphere naming disorders were more pronounced for tool items than animal items.

The neuro-anatomical correlates of category-specific disorders for fruits and vegetables show some features typical of animals (i.e., importance of the anterior and mesial parts of the temporal lobes) and other features typical of artifacts (i.e., left lateralization). These findings, which were recently discussed by Capitani et al. (2009), Gainotti (2010, 2011), and Capitani and Laiacona (2011), are is in keeping with the results of investigations that subjectively evaluated the weight of various kinds of information in the representation of different conceptual categories. In fact, in fruits and vegetables (as in animals) the most relevant sources of information (after vision) are other perceptual data, whereas in all artifact categories they consist of body-related actions and somatosensory data. This explains the critical role of the anterior and mesial parts of the temporal lobes in the representation of all living categories. On the other hand, in the representation of fruits and vegetables (as in those of artifacts, but not animals), specific actions, such as peeling, cutting, and stirring, play an important part, which may account for the shared left lateralization of both artifacts and fruits and vegetables.

### Summary of the main points of this section

Research on the neuro-anatomical correlates of various types of categorical disorders has shown that the cortical areas damaged in patients with a disorder selectively affecting a given category have a critical role in processing the information that primarily contributed to constructing the affected category. Thus, in patients with a category-specific semantic disorder for biological entities, lesions bilaterally affect the anterior parts of the temporal lobes (where the ventral stream of visual processing converges with auditory, olfactory, and gustatory inputs); and in patients with a preferential impairment of the artifact categories lesions usually affect the left-sided fronto-parietal, sensory-motor cortices (where the dorsal stream of visual processing converges with body-related and action-oriented structures). Taken together, both lines of research suggest that body movements and somatosensory information have a major role in the representation of artifacts (mainly known through their manipulation), whereas visual and other perceptual information has a dominant role in the representation of animals and other living things.

## Concluding Remarks

The scope of the present review was ambitious; indeed, it aimed to clarify the nature and format (abstract or sensory-motor) of our conceptual representations. Both the psychological and the anatomo-clinical data summarized in this survey seem to support the sensory-motor (embodied) theory, because they show: (a) that different perceptual and action-related features contribute to the construction of different conceptual categories; (b) that psychological and anatomical data are consistent, because the cortical areas affected in patients with category-specific semantic disorders and activated during tasks involving the same categories play a critical role in processing information that contributed to the construction of the affected category.

These results indicate: (a) that the distinction between biological and artifact categories is not a primary one for the brain and is not due to an “innate” categorical organization of conceptual knowledge, as maintained by Caramazza and co-workers (Caramazza, 1998; Caramazza and Shelton, 1998; Caramazza and Mahon, 2003); (b) that simple dichotomies, such as the “living”/“non-living” distinction or the “SFT” cannot explain the complexity of factors subsuming the brain’s representation of different categories. On the contrary, the assumption that body-related and environmental sources of knowledge experienced through diverse sensory modalities play a different role in the construction of different conceptual categories is consistent with the subjective evaluation of normal subjects and the main functions of cortical areas that have a critical role in the representation of these categories. Nevertheless, the complexity of the experiential factors and brain structures subsuming the brain’s representation of different categories suggests that further investigations are necessary to clarify the advantages and possible limitations of this assumption.

## Conflict of Interest Statement

The author declares that the research was conducted in the absence of any commercial or financial relationships that could be construed as a potential conflict of interest.
